# Directed reprogramming of comprehensively characterized dental pulp stem cells extracted from natal tooth

**DOI:** 10.1038/s41598-018-24421-z

**Published:** 2018-04-18

**Authors:** Rishikaysh V. Pisal, Jakub Suchanek, Richard Siller, Tomas Soukup, Hana Hrebikova, Ales Bezrouk, David Kunke, Stanislav Micuda, Stanislav Filip, Gareth Sullivan, Jaroslav Mokry

**Affiliations:** 1 Department of Histology and Embryology, Faculty of Medicine in Hradec Kralove, Charles University, Simkova 870, 500 03 Hradec Kralove, Czech Republic; 2 Department of Dentistry, Faculty Hospital in Hradec Kralove and Faculty of Medicine in Hradec Kralove, Charles University, Simkova 870, 500 03 Hradec Kralove, Czech Republic; 3 Norwegian Center for Stem Cell Research, University of Oslo, 0317 Oslo, Norway; 4 Department of Molecular Medicine, Institute of Basic Medical Sciences, University of Oslo, 0317 Oslo, Norway; 5 Department of Biophysics, Faculty of Medicine in Hradec Kralove, Charles University, Simkova 870, 500 03 Hradec Kralove, Czech Republic; 6 Department of Pharmacology, Faculty of Medicine in Hradec Kralove, Charles University, Simkova 870, 500 03 Hradec Kralove, Czech Republic; 7 Department of Oncology and Radiotherapy, Faculty of Medicine in Hradec Kralove, Charles University, Simkova 870, 500 03 Hradec Kralove, Czech Republic; 8 Institute of Immunology, Oslo University Hospital-Rikshospitalet, PO Box 4950 Nydalen, Oslo, 0424 Norway; 9 Hybrid Technology Hub - Centre of Excellence, Institute of Basic Medical Sciences, University of Oslo, Blindern, 0317 Oslo, Norway; 10 Department of Pediatric Research, Oslo University Hospital, 0424 Nydalen, Norway

## Abstract

The aim of this study was to extensively characterise natal dental pulp stem cells (nDPSC) and assess their efficiency to generate human induced pluripotent stem cells (hiPSC). A number of distinguishing features prompted us to choose nDPSC over normal adult DPSC, in that they differed in cell surface marker expression and initial doubling time. In addition, nDPSC expressed 17 out of 52 pluripotency genes we analysed, and the level of expression was comparable to human embryonic stem cells (hESC). Ours is the first group to report comprehensive characterization of nDPSC followed by directed reprogramming to a pluripotent stem cell state. nDPSC yielded hiPSC colonies upon transduction with Sendai virus expressing the pluripotency transcription factors *POU5F1, SOX2, c-MYC* and *KLF4*. nDPSC had higher reprogramming efficiency compared to human fibroblasts. nDPSC derived hiPSCs closely resembled hESC in terms of their morphology, expression of pluripotency markers and gene expression profiles. Furthermore, nDPSC derived hiPSCs differentiated into the three germ layers when cultured as embryoid bodies (EB) and by directed differentiation. Based on our findings, nDPSC present a unique marker expression profile compared with adult DPSC and possess higher reprogramming efficiency as compared with dermal fibroblasts thus proving to be more amenable for reprogramming.

## Introduction

Adult stem cells have an edge over somatic cells in terms of being an efficient starting cell source for reprogramming^1^. This is because they have relatively fast cell cycle kinetics, plasticity and endogenously express a subset of pluripotency factors, albeit at lower levels compared to embryonic stem cells. As opposed to somatic cells, adult stem cells are relatively undifferentiated with pronounced plasticity^2,3^. Various studies have pointed out that adult stem cells are more amenable and efficient in reprogramming than somatic or mature cells^4–9^. In sharp contrast to differentiated cells, adult stem cells have more reprogramming enhancers than barriers. Expression of stemness related genes, permissible chromatin state, a decreased level of barriers (e.g. TGF-β and MAP kinase pathways) and increased levels of genetic and epigenetic facilitators (e.g. KDM2B)^10^ are some of the intrinsic features that act as reprogramming enhancer. In summary, expression of pluripotency factors, lack of lineage-specific gene expression and permissible chromatin state makes adult stem cells more efficient to reprogramming over differentiated cells.

Examples of stem cells that were successfully reprogrammed are as follows. CD133^+^ stem cells were efficiently reprogrammed to pluripotent stem cells state using just two factors namely *POU5F1* and *SOX2*^5^. While, ectopic expression of *POU5F1* was sufficient to reprogram neural stem cells as they endogenously expressed high levels of *SOX2*, *c-MYC* and *KLF4*^6,11^. Examples of few other stem cells lines, which were successfully reprogrammed are adipose stem cells^12^, dental pulp stem cells (DPSC)^13^, hematopoietic stem cells and endometrial stem cells^4,14^. All these stem cells were more amenable to reprogramming than fibroblasts.

There are a number of distinctive features of DPSC that make them an attractive candidate for dedifferentiation or reprogramming. Primarily, they are multipotent stem cell in nature and express a myriad of markers including STRO-1, vimentin, CD29, CD44, CD73, CD90, CD166, as well as the stem cell markers nestin and nucleostemin^15^. It is advantageous to use stem cells as a starting cell point for reprogramming because the efficiency of reprogramming is higher as compared to dermal fibroblasts or other terminally differentiated tissue types^16^. In addition, the risk of exomic mutations due to aging is minimal in DPSC^17^ and the acquisition of DPSC is a relatively less invasive procedure.

Natal teeth are teeth that are present at birth, the incidence of natal and neonatal teeth ranges from 1:2,000 to 1:3,500^18^. Natal teeth are extracted at the earliest time-point possible because they may induce deformity or mutilation of the tongue leading to dehydration, inadequate nutrient uptake by the infant followed by growth retardation^19^. The extracted teeth are normally considered as biological waste. However, dental pulp present within the tooth is a rich source of stem cells and this repertoire of stem cells can potentially be used for regenerative medicine^20^.

The recently described Sendai virus platform for human induced pluripotent stem cell (hiPSC) generation is an ideal vector system for delivering reprogramming factors because of the following reasons: (i) the virus replicates in the form of negative-sense single-stranded RNA in the cytoplasm of infected cells and it does not integrate into the host genome^21^; (ii) it efficiently delivers exogenous genes in a wide spectrum of host cell species and tissues^22^; (iii) it shows low cytotoxicity upon infection^23,24^; and (iv) allows high-level expression of the delivered exogenous genes^25^.

In the current study, we sought to isolate, characterise and reprogramme natal dental pulp stem cells (nDPSC). nDPSC were initially characterized by detecting the expression of cell surface markers using flow cytometry. Then the nDPSC derived hiPSC clones were characterised to confirm complete reprogramming to pluripotency by performing immunostaining, gene expression analysis and embryoid body based spontaneous differentiation and directed differentiation to all three germ lineages.

## Results

### nDPSC express mesenchymal stem cell markers

**Figure 1:**
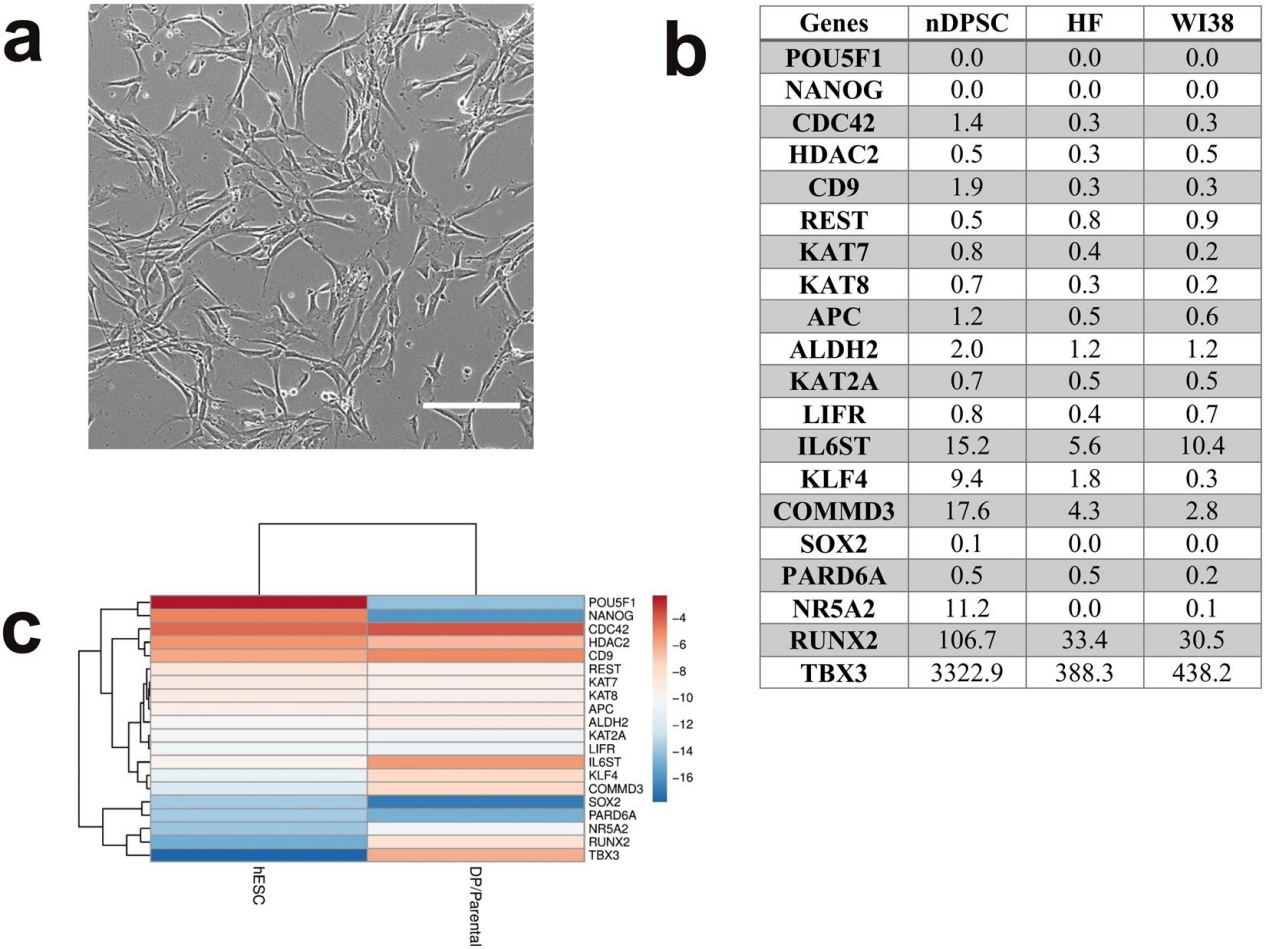
Morphology and gene expression profile of nDPSC. (**a**) Morphology of nDPSC under phase contrast microscope. (**b**) Comparison of expression of 20 pluripotency genes between nDPSC and two cell lines of human fibroblasts. Values represent fold change. 2^−∆∆CT^ formula was used to calculate the fold change and hESC was used as calibrator sample. (**c**) RT-qPCR expression profiling of pluripotency genes in hESC and nDPSC. The heat map was generated by presenting −∆Ct (C_T gene_ − C_T ACTB_) values of each gene. Red colour and lower value indicates higher expression. Scale bar = 200 µm.

**Figure 2:**
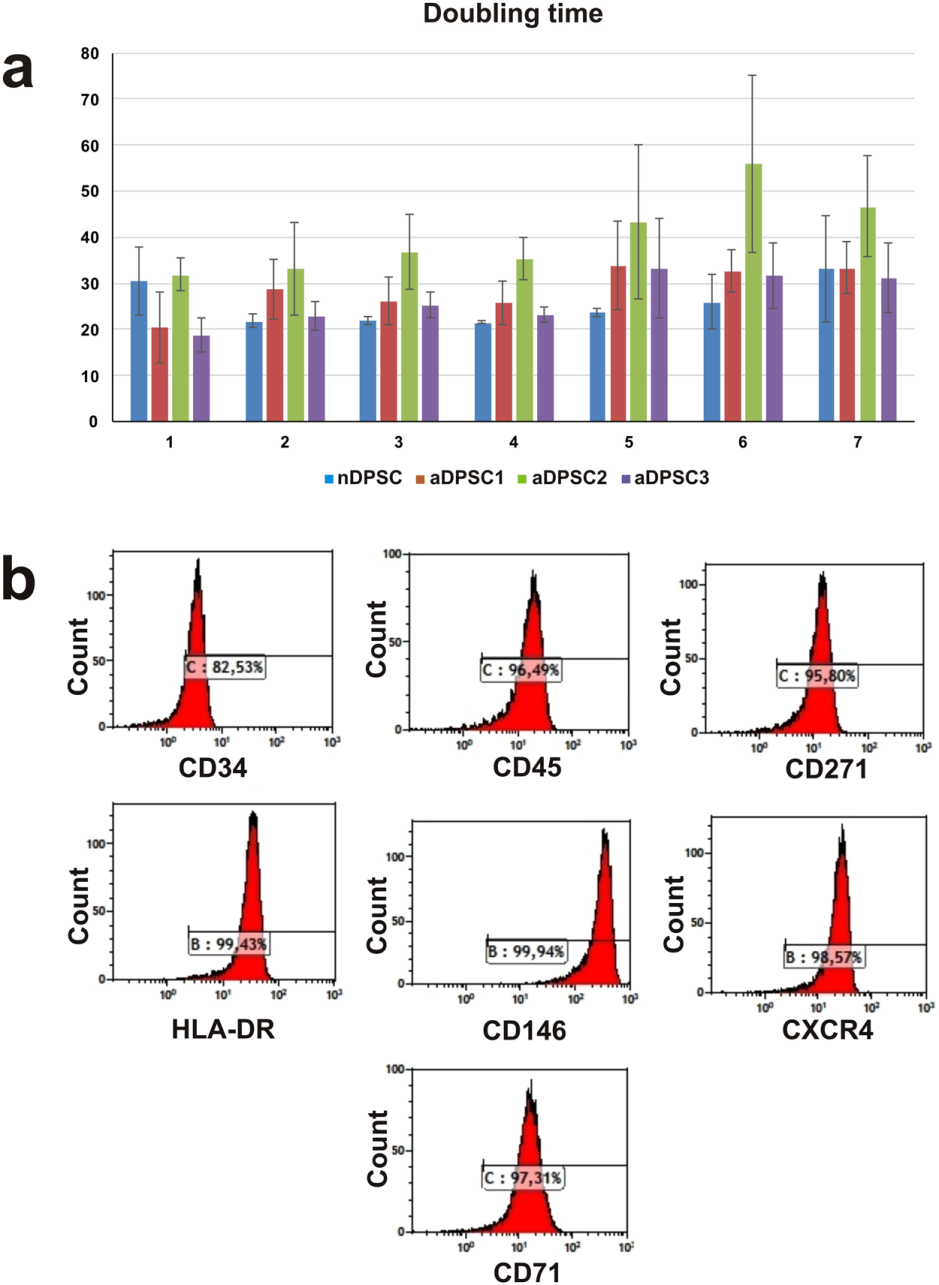
Growth pattern and flow cytometry data. (**a**) Comparison of doubling time between nDPSC and adult DPSC. Doubling time of nDPSC is compared with three adult DPSC cell lines during initial passages. Data are presented as the average +/− standard deviation; n = 3. (**b**) Flow cytometry histograms representing expression of markers characteristic to nDPSC; these markers are not expressed or are expressed at low levels in adult DPSC. nDPSC exhibited high expression of CD34, CD45, CD271, CD71, HLA-DR, CD146 and CXCR4 markers.

Natal DPSCs (nDPSC) were isolated from a natal tooth extracted from an 11-day-old baby girl using our previously described procedure^26^. The derived nDPSC cells exhibited a fibroblastic morphology when observed under phase contrast microscopy (See Fig. 1a). Doubling time of nDPSC during initial passaging (i.e. from 2 to 6) ranged from 21 to 25 hr while that of adult DPSC cell lines ranged from 22 to 58 hr (See Fig. 2a). Compared to adult DPSC cell lines, nDPSC had a faster doubling time from passage 2 to 6 (See Fig. 2a). Comparative gene expression analysis between human embryonic stem cells (hESC) and nDPSC revealed that while the expression pattern of 17 genes associated with pluripotency were similar (See Fig. 1c), however the core pluripotency factors such as *POU5F1, SOX2* and *NANOG* were not highly upregulated in nDPSC (See Fig. 1c). Expression of above mentioned 20 pluripotency genes of nDPSC were also compared to human dermal fibroblasts (HF) and WI38 human embryonic lung fibroblasts (See Fig. 1b). *IL6ST*, *KLF4*, *COMMD3*, *NR5A2*, *RUNX2* and *TBX3* genes were highly expressed in nDPSC as compared to other genes. 2^−∆∆CT^ formula was used to calculate the fold change and hESC was used as calibrator.

Flow cytometry (FC) results confirmed expression of cell surface markers indicative of mesenchymal stem cells (MSC) such as CD44, CD73, CD271, CD90, CD105, CD166, CD45 and CD10. Apart from MSC markers, nDPSC also expressed markers related to hematopoietic stem cells (HSC) such as CD34, CXCR4, CD71, CD45 and CD10. Other markers expressed were CD222 and HLA-DR (See Table 1 and Fig. 2b). This indicates that nDPSC are multipotent and we predicted highly amenable to reprogramming towards pluripotency^27^.

**Table 1:** Comparative analysis of various markers expressed by nDPSC and adult DPSC.

Markers	nDPSC	adult DPSC*
CD34	+++	−
CD45	+++	−
CD271	+++	+
CD71	+++	+
HLA-DR	+++	−
CD146	+++	−
INF-beta	+++	Variable
CXCR4	+++	++
CD29	+++	+++
CD105	+++	+++
CD222	+++	+++
CD166	+++	+++
CD44	+++	+++
CD90	+++	+++
CD10	+++	+++
CD13	+++	+++
CD73	+++	+++

Flow cytometry analysis was performed to characterize nDPSC. Cells were grown on media as mentioned by Karbanova, J *et al*.^29^. Signal intensity:+++ = strong; ++ = medium; + = weak; − = lack of marker expression. *References^28–32^.

nDPSC expressed various markers characteristic of MSC and HSC which might indicate that these cells are relatively immature progenitor cell population and that the expression profile of nDPSC is unique to this cell type, as the majority of markers excluding the MSC specific markers, are not normally expressed by adult DPSC (See Table 1)^27–31^.

### Generation of hiPSC from nDPSC

**Figure 3:**
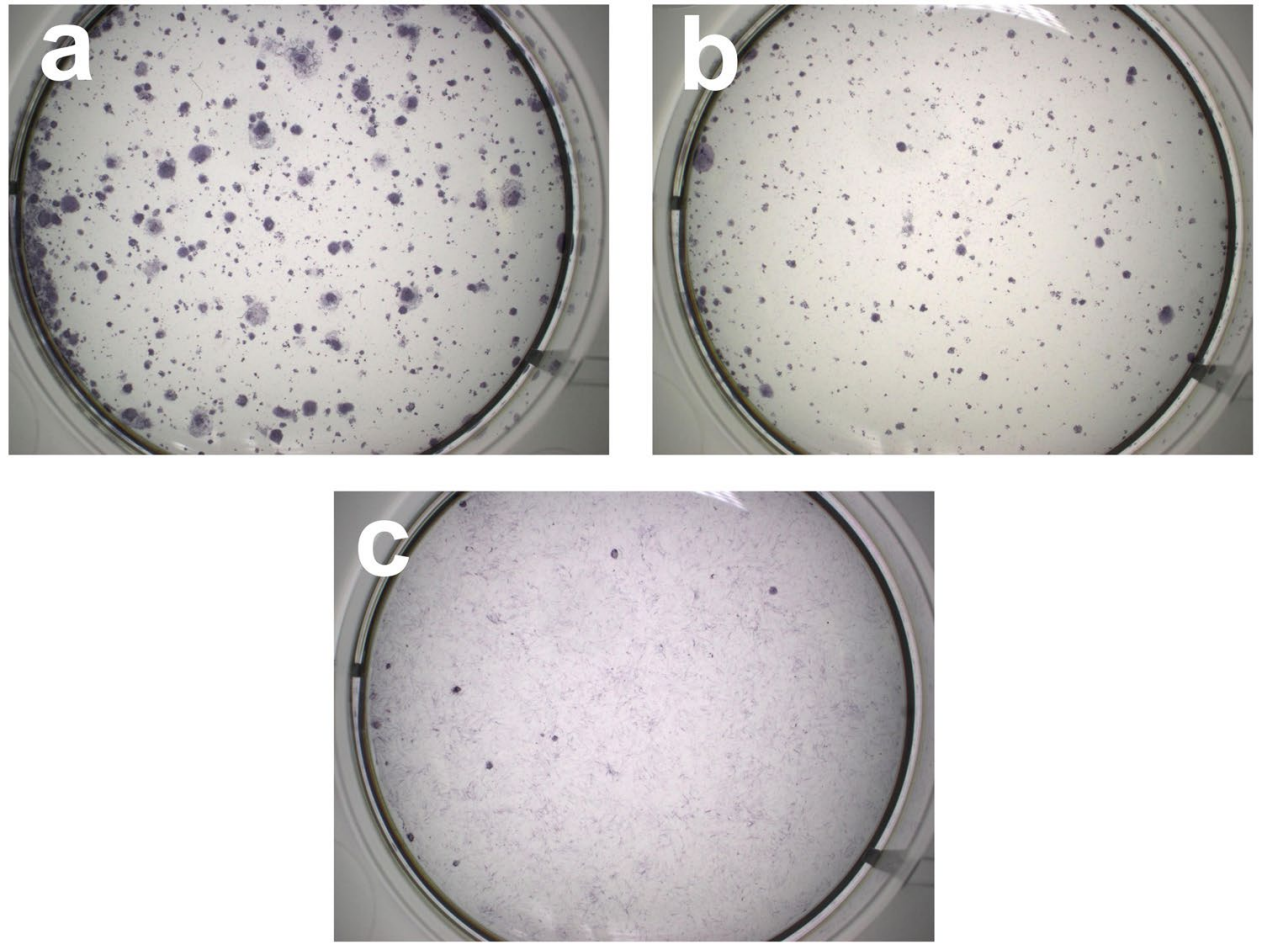
Alkaline phosphatase (AP) staining to calculate reprogramming efficiency. Reprogramming efficiency was calculated by counting number of AP positive colonies after emergence of iPSC colonies following transduction of Sendai reprogramming vectors. Each 6 well plate was seeded with 75000 cells on 7^th^ day post transduction. nDPSC (**a**) showed 966 AP positive colonies, while dermal fibroblasts (**b**) showed 550 colonies and WI38 lung fibroblasts (**c**) showed 14 colonies.

hiPSC colonies began to emerge on 13^th^ day post transduction. On 7^th^ day post transduction, 75000 cells from each cell line were plated on recombinant human vitronectin (rhVTN) coated plates to determine reprogramming efficiency using alkaline phosphatase (AP) staining. nDPSC cells were stained for AP on 18^th^ day post transduction (See Fig. 3a), human fibroblasts (HF) on 25^th^ day (See Fig. 3b) and WI38 on 27^th^ day (See Fig. 3c). In nDPSC, 966 colonies stained positive, 550 in HF and 14 in WI38. Hence, reprogramming efficiency of nDPSC was the highest with 1.3% followed by HF with 0.73% and finally WI38 with 0.018%. In conclusion, nDPSC were more efficient and reprogrammed earlier than the fibroblasts.

**Figure 4:**
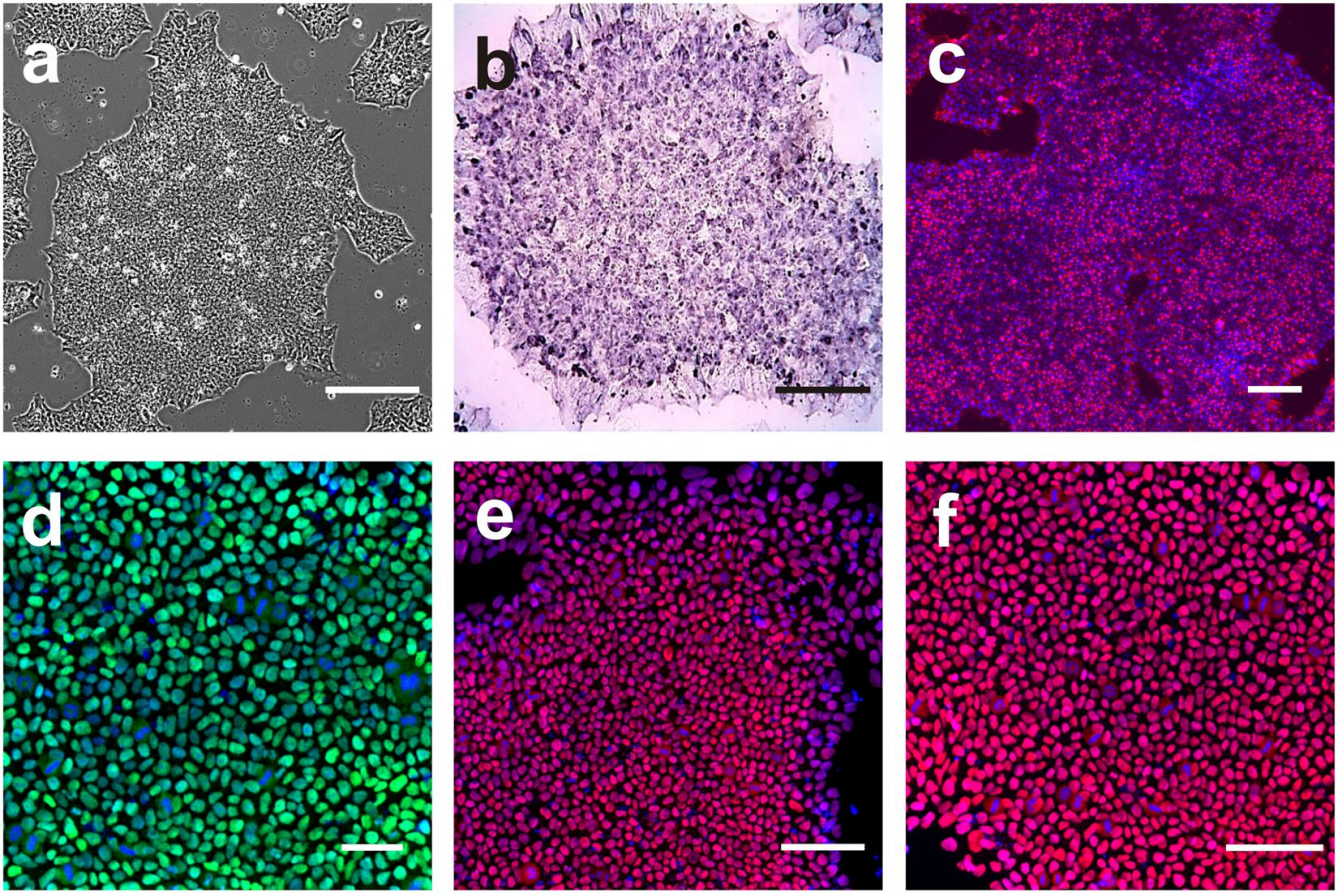
(**a**) nDPSC derived hiPSC. Image of nDPSC derived hiPSC with typical hES like morphology. (**b**) Colorimetric detection of alkaline phosphatase. (**c**–**f**) Immunocytochemistry against (**c**) SSEA-4, (**d**) POU5F1, (**e**) SOX2, and (**f**) NANOG. Nuclei were counterstained with DAPI. Images are shown as overlap of the two channels (**c**–**f**). Scale bar = 200 µm.

hiPSC colonies were manually picked once they attained an appropriate size, which was around day 18. hiPSC clones were expanded on recombinant human rhVTN coated plates and maintained in Essential 8 medium (E8). In total 35 individual clones were isolated. hiPSC clones formed tightly packed, flat colonies (See Fig. 4a). hiPSC clones showed significant up-regulation of the critical pluripotent cell markers such as alkaline phosphatase and stage-specific embryonic antigen (SSEA-4) and core pluripotency factors *POU5F1*, *SOX2* and *NANOG* (See Fig. 4).

### Comparative gene expression analysis between nDPSC derived hiPSC, fibroblast derived hiPSC and hESC

For gene expression analysis, a critical set of 83 genes was assessed. These genes were broadly classified into three groups as follows: pluripotency markers comprising of 52 genes; early differentiation markers with 18 genes; and somatic cell markers with 13 genes (See Table S1). For constructing heat map −∆C_T_ (C_T gene_ − C_T ACTB_)^32^ values of six samples i.e. hESC (CCTL 4), DP/iP/C3, DP/iP/C28, DP/iP/C4, HF/iP/C8 and WI38/iP/C5 were used. DP/iP/C3, DP/iP/C28 and DP/iP/C4 are hiPSC clones derived from nDPSC, HF/iP/C8 from human dermal fibroblasts and WI38/iP/C5 from human embryonic lung fibroblasts.

**Figure 5:**
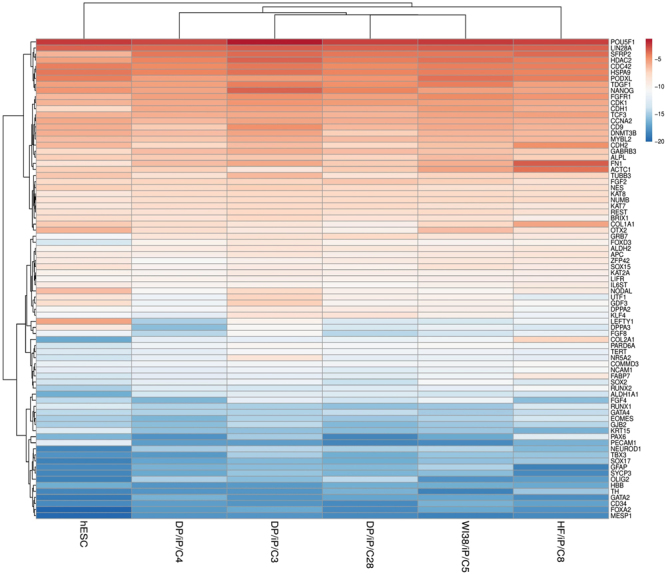
RT-qPCR expression profiling of pluripotency genes in hESC, nDPSC hiPSC clones (DP/iP/C3, DP/iP/C28 and DP/iP/C4), HF hiPSC clone (HF/iP/C8) and WI38 hiPSC clone (WI38/iP/C5). The heat map was generated by presenting −∆Ct (C_T gene_ - C_T ACTB_) values of each gene. Red colour and lower value indicates higher expression. Parameters for generating the heat map are as follows: No scaling was applied to rows and rows were clustered using Euclidean distance and complete linkage. Data are expressed as means of technical duplicates.

The heat map (See Fig. 5) was sub-divided into three subgroups based on gene expression: high expression, medium expression and low expression. The top showing 32 genes were highly expressed i.e. up to *OTX2*; the next 26 genes in the map had medial expression i.e. from *GRB7* up to *RUNX2* and last 21 genes had low expression. In the high expression subgroup, except for five genes (*CDH1, NES, FN1, TUBB3*, and *COL1A1*) all belonged to the pluripotency marker group. Out of these five genes, *CDH1* and *NES* are from the somatic cell markers group while *FN1, TUBB3*, and *COL1A1* are from the early differentiation markers group. Upregulation of *CDH1* in iPSC clones is very critical because, *CDH1* plays crucial role in development^33^ and in mesenchymal to epithelial transition (MET) during reprogramming^34^. In medium expression subgroup, all genes are from the pluripotency marker group with the exception of *FABP7*, which is from the somatic cell markers group. The low expression subgroup is mainly comprised of early differentiation markers and markers of somatic cells, with the following exceptions, *TBX3*, which is from the pluripotency marker group.

In summary, all the pluripotency marker genes are highly expressed in nDPSC derived hiPSC clones, HF hiPSC clones, WI38 hiPSC clones and hESC, with the abovementioned exceptions, while the early differentiation and somatic cell marker genes have low expression levels as expected for *bona fide* hPSCs.

From a glance at the heat map, we can say that the overall gene expression patterns between nDPSC derived hiPSC clones (DP/iP/C3, DP/iP/C28 and DP/iP/C4), HF hiPSC clone (HF/iP/C8) embryonic lung fibroblasts hiPSC clone (WI38/iP/C5) and hESC are very similar with exception of *LEFTY1 and FOXD3* genes where the pattern is dissimilar. The pattern of expression is exactly opposite for *LEFTY1* and *FOXD3. LEFTY1* is highly expressed in hESC while in all hiPSC clones it is expressed at low to medium level. Expression level of *FOXD3* is lower in hESC as compared to all hiPSC clones, where it is expressed at medium level.

**Figure 6:**
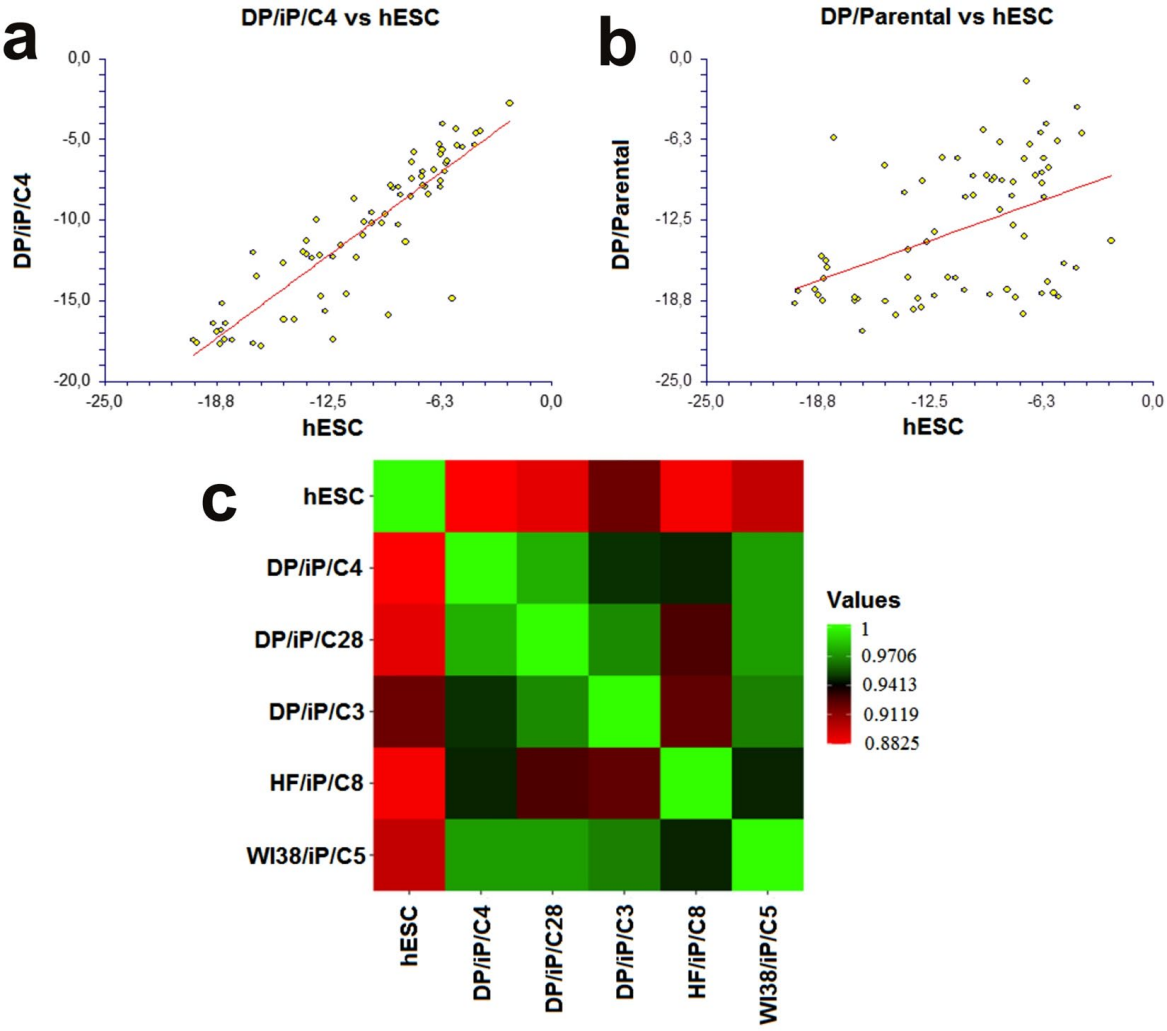
Gene expression patterns were compared between DP/iP/C4 vs hESC and DP/Parental vs hESC using linear regression plot (**a** & **b**). −∆Ct (C_T gene_ − C_T ACTB_) values were used for constructing the plot. The line indicates the trend of the graph. Data are expressed as means of technical duplicates. Statistical correlation analysis shows that there is statistically significant correlation in gene expression pattern between DP/iP/C4 and hESC (*P* < 0.0001) with coefficient of correlation 0.8854. This correlation is regarded as a strong correlation. There is also a statistically significant co-relation between hESC and DP Parental (*P* = 0.0001) with coefficient of correlation 0.4571 and this is categorized as moderate correlation. (**c**) Correlation analysis between hESC and hiPSC clones. Correlation coefficient values are represented in form of heat map in pairwise matrix. Light red colour indicates lowest value while light green indicates highest value.

The overall expression patterns between nDPSC hiPSC clones, hESC and HF hiPSC clones is very similar (See Fig. 5). Linear regression plot shows that there is strong correlation between hESC and one of the clones of nDPSC iPSC (See Fig. 6a). While there is moderate correlation between nDPSC and hESC; from these results we can speculate that, nDPSC have pluripotent stem cell like characteristics but have not attained pluripotency to the level of hESC (See Fig. 6b)^27^. nDPSC derived hiPSC clones are highly similar to hESC with the correlation coefficient greater than 0.88. Correlation of nDPSC derived iPSC clones is maximal within themselves and with HF hiPSC clones followed by hESC (See Fig. 6c).

### Differentiation of nDPSC derived hiPSC by embryoid body formation and directed differentiation

**Figure 7:**
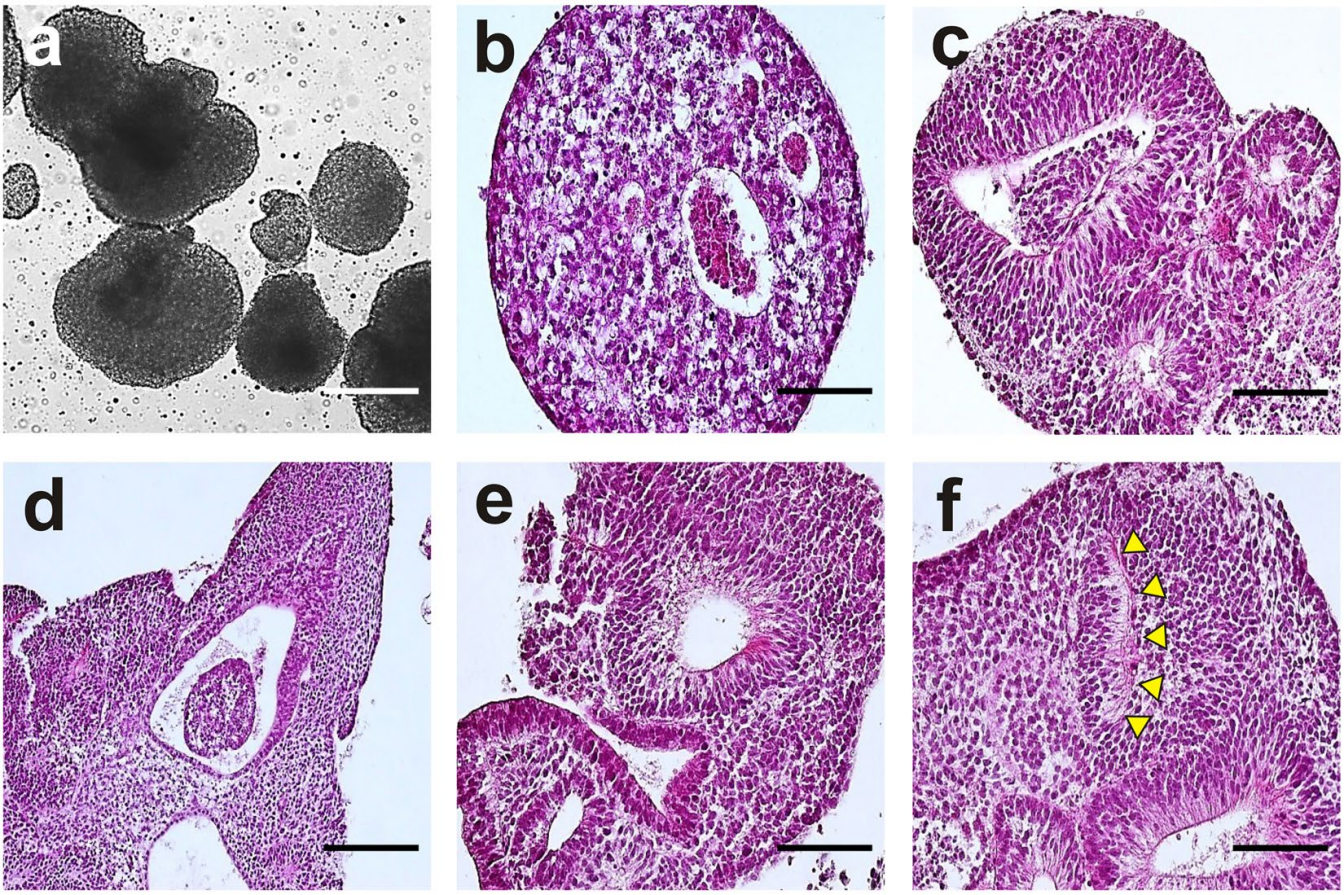
Embryoid body-mediated differentiation of nDPSC derived hiPSC. (**a**) EB derived from nDPSC derived hiPSC at day 5. (b-f) Haematoxylin and eosin stained embryoid bodies revealing various structures formed within them including (**b**) blood islands, (**c**) neural rosettes, (**d**) gut-like epithelium, (**e**) external covering epithelium, and (**f**) optic cup-like structure; arrows show the location of the optic cup primordium. Scale bar = 200 µm.

**Figure 8:**
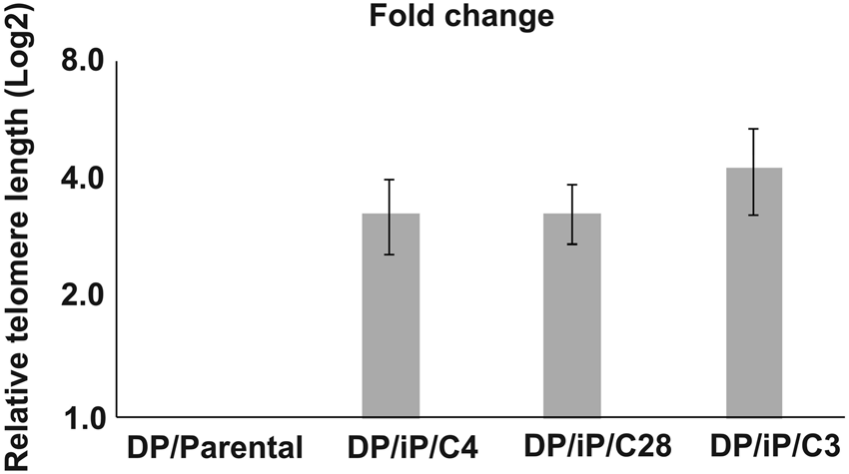
Relative telomere analysis was performed using qPCR. The graph represents log_2_ fold change of relative telomere length in parental cell DP/Parental and nDPSC-derived hiPSC (DP/iP/C4, DP/iP/C28 and DP/iP/C3). Data is presented as the average +/− standard deviation; n = 3.

The differentiation potential of nDPSC derived hiPSCs was verified by generating embryoid bodies (EB) in order to induce spontaneous differentiation. EB were cultured for 21 days after which they were harvested and processed via paraffin embedding, followed by sectioning for analysis. Haematoxylin and eosin staining of EB sections revealed various structures characteristic of the three germ layers (See Fig. 7). Blood islands (mesoderm) (See Fig. 7b), neural rosettes (ectoderm) (See Fig. 7c), gut like epithelium (endoderm) (See Fig. 8d), covering epithelium (ectoderm) (See Fig. 7e) and optic plate (ectoderm) (See Fig. 7f) like structures were a few representative structures visible after staining the sections.

Immunohistochemistry of sectioned EB showed strong positivity for β-III tubulin (a marker of ectoderm), MAP2 (ectoderm), nestin (ectoderm), vimentin (mesoderm marker) and pan cytokeratin (endoderm marker) (See Supplementary Fig. S1). These results show that nDPSC-derived hiPSC have the potential to differentiate into the three germ layers, *in vitro*, during spontaneous differentiation in EB.

In addition, iPSC clones obtained from nDPSC, HF and WI38 cell lines were individually differentiated using directed differentiation approaches into neuroepithelium (ectoderm), cardiomyocytes (mesoderm) and hepatic endoderm (endoderm). Presence of neuroepithelium was confirmed by observing characteristic morphology of the differentiated cells and by RT-qPCR analysis (See Fig. S3). Beating cell clusters confirmed existence of cardiomyocytes (See supplementary videos). Cobblestone morphology together with immunostaining and qPCR results confirmed strong expression of hepatic markers, thus assuring presence of hepatic endoderm (See Fig. S2).

Morphological and immunohistochemistry results highlight that nDPSC derived hiPSC had attained pluripotency as they were capable of differentiation to all three lineages.

### Telomere length and karyotype analysis

Telomere length analysis was carried out by qPCR according to the method described by Cawthon^35^. When compared to the parental nDPSC line, the relative telomere length increased by three-fold in DP/iP/C4 and DP/iP/C28 clones respectively (See Fig. 8) and by four fold in the DP/iP/C3 clone. DP/iP/C4 and DP/iP/C28 were established clones (passage 12) while DP/iP/C3 clone was at a relatively low passage (passage 7); this might have contributed to the observed differences in telomere lengths^36–38^.

Karyotype analysis of nDPSC derived hiPSC at passage 12 demonstrated that they had a normal karyotype of 46, XX without aneuploidy or polyploidy (See Supplementary Fig. S4).

Reprogramming of nDPSC led to a threefold increase in telomere lengths as compared to the starting parental cell line with a normal karyotype.

## Discussion

Dental pulp harbours a stem cell population commonly referred to as DPSC, which possess properties similar to generic MSC^39^. Both adult DPSC and nDPSC had a similar expression profile for CD10, CD13, CD29, CD44, CD73, CD105, CD90 and CD166 but not for CD146, CXCR4, CD271, CD71, CD34, CD45 and HLA-DR. Adult DPSC expressed CD146^moderate^, CXCR4^weak^, CD271^weak^, CD71^weak^, CD34^−^, CD45^−^ and HLA-DR^−^ while nDPSC showed high positivity for all the markers^27–31^. It has been previously reported that nDPSC have an absence of CD34, CD45, CD71 and HLA-DR markers^40,41^. Surprisingly, our nDPSC were highly positive for CD34, CD45, CD71 and HLA-DR markers. Our finding demonstrates that the isolated population of nDPSC expressed HSC markers and HLA class II molecules in addition to MSC markers, which is a unique expression profile as compared to both adult DPSC and counter to the report of Akpinar and colleagues^40^.

Gene expression analysis revealed that nDPSC expressed 17 out of 52 pluripotency genes at levels equivalent to hESC. Overall gene expression analysis showed a moderate correlation between nDPSC and hESC. Previous studies have shown that adult DPSC express a number of pluripotency markers including *POU5F1, NANOG, c-MYC, SOX2*, stage specific embryonic antigens (SSEA-3, SSEA-4), and tumour recognition antigens (TRA-1–60 and TRA-1–81)^40,42^. However, the expression levels are significantly lower compared with the values observed in hESC^43^. From these studies and our data, we could speculate that promoter regions of pluripotency genes in nDPSC may have a low methylation status and favourable transcriptional as well as epigenetic state for reprogramming.

*NR5A2* is medially expressed in nDPSC and the level of expression is higher as compared to hESC. Jian *et al*., had previously shown that, *NR5A2* enhances kinetics of *OSKM* mediated reprogramming and *NR5A2* functions synergistically with *SOX2* and *KLF4* to replace exogenous *POU5F1* to mediate the successful reprogramming of murine somatic cells to pluripotent cells^44^. In addition, increase in *NR5A2* expression directly causes an increase in *NANOG* expression^44^. An important point to be noted here is that, KLF4 is highly expressed in nDPSC and this same factor is used for reprogramming. For future experiments, it would be interesting to reprogramme nDPSC by eliminating *KLF4* from the cocktail of transcription factor used for reprogramming. Another gene that grabs attention is *IL6ST*, which is an interleukin 6 signal transducer. This receptor activates Ras-ERK, JAK1-STAT3, and PI3K pathways^45^. Activation of STAT3 has been shown to enhance proliferation and stemness in glioma-associated-human MSCs^46^. Moreover, Hingwei *et al*., showed that STAT3 activity can reprogramme conventional (primed) hPSC to naive-like hPSC^47^. In case of nDPSC, they show high positivity for IL6 (IFN-beta) (flow cytometric data) as well as high expression of its corresponding receptor *IL6ST*. In summary, we can say that nDPSC are immaturely differentiated and could be classified as naïve multipotent stem cells.

Large and numerous reprogrammed colonies appeared on the 18^th^ day post transduction, which indicates that nDPSC underwent reprogramming both rapidly and efficiently^48^. Reprogramming efficiency of nDPSC (1.3%) reported in this article is significantly higher than the efficiencies 0.1%, 0.09% and 0.24%, which are reported in previously published articles on DPSC^13,49,50^.

Based on the time of upregulation during reprogramming, pluripotency gene markers are classified into early upregulated group and late upregulated group^51^. *LEFTY1* is expressed during the early phase of reprogramming while *FOXD3* is upregulated at later stage^51^. *FOXD3* and *LEFTY1* have been reported to be crucial in maintaining pluripotency^51^. During reprogramming, the early upregulated group is activated between 10 to 20 days and the expression of these genes lasts beyond day 30^51^. However, late upregulated group is expressed only in a few of the colonies at day 30^51^. Colonies expressing late upregulated group can be used for isolating colonies because such colonies have high probability of becoming fully reprogrammed hiPSC^51^. This discussion explains the results observed in nDPSC derived hiPSC and hiPSC clones derived from HF and WI38 cell lines. Further comparative studies need to be performed in order to reveal the overall reprogramming competency of DPSC isolated from donors of different age groups and gender. The results presented here demonstrate that nDPSC are an efficient starting cell type for reprogramming.

To our knowledge, this is the first report for the complete characterization and reprogramming of nDPSC. In addition, nDPSC present a unique and beneficial gene and surface marker expression as well as superior proliferation capacity when compared to their adult counterparts. These characteristics make them ideal starting material for the generation of hiPSC.

## Materials and Methods

### Cell isolation, culture and reprogramming

A natal tooth was extracted from an 11 day old baby girl. Isolation and expansion of nDPSC was performed as previously described^26,28^. During the initial phase, nDPSC were cultured in media supplemented with human platelet rich plasma^26^.

For studying doubling time (DT), natal DPSC and adult DPSC were seeded at 5000 cells/cm^2^. After reaching 70% confluence, cells were harvested and were counted using Vi-CELL Cell Counter. The experiment was performed in triplicates for each time point. Doubling time was calculated using following formula:

$$\frac{({\mathrm{Log}}_{10}(2)\times {\rm{culture}}\,{\rm{time}}\,{\rm{in}}\,{\rm{hour}})}{({\mathrm{log}}_{10}({\rm{number}}\,{\rm{of}}\,{\rm{harvested}}\,{\rm{cells}})-{\mathrm{log}}_{10}({\rm{number}}\,{\rm{of}}\,{\rm{seeded}}\,{\rm{cells}}))}$$

For reprogramming, we employed the CytoTune-iPS 2.0 Sendai Reprogramming Kit (Life Technologies). nDPSC were maintained as previously described by Karbanova and colleagues^28^ prior to reprogramming as well as three days post transduction. The medium in which nDPSC were cultured, will henceforth be referred as DPSC medium. After this period, cells were cultured in DPSC medium but without growth factors for next five days. On seventh day, cells were detached using TrypLE select cell dissociation reagent (Life Technologies). Cells were counted and were plated at 5 × 10^5^ and 1 × 10^5^ densities on rh-VTN (Life Technologies) coated 100 mm plates. Following trypsinization, cells were cultured in DPSC medium without growth factors for 24 hrs. From eighth day onwards, cells were cultured in E8 (Life Technologies) medium and the spent medium was replaced every day. hiPSC clones were manually picked and subsequently expanded on rh-VTN coated plates with Essential 8 medium. For passaging, hiPSC clones were washed twice with DPBS (Life Technologies; Cat no. 14190169) without calcium and magnesium and then incubated with 0.5 mM EDTA at 37 °C for approximately 3 min^52^. The cells were detached from the well by gentle pipetting, ensuring that the colonies remained in chunks. The split ratio was routinely 1:3.

In addition to nDPSC, we used adult human fibroblasts and embryonic lung fibroblasts WI-38 (ATCC) for reprogramming. WI-38 fibroblasts were cultured in MEM (Life Technologies) media supplemented with 10% FBS (Sigma) while adult human fibroblasts were cultured in DMEM (Life Technologies) media supplemented with 10% FBS (Sigma). Adult human fibroblasts were isolated from skin biopsy of female patient undergoing cosmetic surgery, using a previously described protocol^53^.

### Flow cytometry

nDPSC were harvested by trypsin/EDTA treatment for 5 min at 37 °C. After inactivation of trypsin and centrifugation (5 min at 300 *g*), 100 µl of the cell suspension (10^5^ cells) was incubated for 30 min at room temperature in the dark with the following fluorochrome-conjugated antibodies: anti-CD10 (eBiosciences; Clone: CB-CALLA), anti-CD13 (eBiosciences; Clone: WM-15), anti-CD29 (Chemicon, Clone: TDM29), anti-CD34 (Biolegend; Clone: 4H11), anti-CD44 (Caltag; Clone: MEM-85), anti-CD45 (Caltag; Clone: HI30), anti-CD71 (Biolegend; Clone: MEM-75), anti-CD73 (BD Pharming; Clone: AD2), anti-CD90 (Chemicon; Clone: F15-42-1), anti-CD105 (Caltag; Clone: SN6), anti-CD146 (Millipore; Clone: P1H12), anti-CD166 (Beckman; Clone: 3A6), anti-CD222 (eBiosciences; Clone: MEM-238), anti-CD271 (Biolegend; Clone: ME20.4), anti-CXCR4 (Caltag; Clone: 12G5), anti-INF-beta (INFsource; Clone: MMHB-3) and anti-HLA-DR (Caltag; Clone: Tu36). After washing with PBS, at least 10,000 events were acquired using a Beckman Coulter FC500 flow cytometer. Instrument settings and gating strategies were established using isotype controls, and data were analysed using Kaluza software (Beckman Coulter). All experiments were carried out in duplicates.

### Alkaline phosphatase staining and immunocytochemistry

Alkaline phosphatase staining was performed using a colorimetric method. hiPSC were incubated with NBT and BCIP substrates (nitro-blue tetrazolium and 5-bromo-4-chloro-3′-indolyphosphate respectively), which yielded an intense, insoluble black-purple precipitate on reaction with alkaline phosphatase. For immunofluorescence staining, hiPSC were grown on rh-VTN coated 12-well plates. After reaching the desired confluency, the medium was removed by aspiration and the cells were washed with DPBS-T (DPBS (Life Technologies; cat. no. 14190169) containing 0.1% Tween 20), followed by fixation with 4% paraformaldehyde for 10 min at room temperature. After washing with DPBS-T, the cells were permeabilized using 0.5% Triton X-100 for 10 min at room temperature. The cells were washed again with DPBS-T, after which the cells were then blocked with DPBS supplemented with 5% normal goat (Jackson ImmunoResearch) or donkey serum (Jackson ImmunoResearch) and incubated at room temperature for 30 min. Without washing, cells were incubated with primary antibodies overnight at 4 °C. The following primary antibodies were used: anti-SSEA4 (1:20, MC-813-70 (SSEA-4); Developmental Studies Hybridoma Bank), anti-POU5F1 (OCT-4) (1:100, sc-8629; Santa Cruz), anti-SOX2 (1:100, ab92494; Abcam) and anti-NANOG (1:100, PA1-097X; Life Technologies). The primary antibody was removed and the cells were washed with DPBS-T, followed by incubation with secondary antibodies for 45 min at room temperature, in the dark. The following secondary antibodies were used: cyanine 3 (Cy3) (Jackson ImmunoResearch) conjugated and Alexa488 (Jackson ImmunoResearch) conjugated IgG. The nuclei were counterstained with DAPI (Sigma) for 5 min.

Fluorescent images were captured using a Nikon Eclipse Ti-E inverted fluorescence microscope equipped with Nikon DS-Qi2 camera. Images were analysed and processed using NIS-Elements for advanced research (NIS-Elements AR) software.

### RNA isolation and reverse transcription for SYBR RT-qPCR

Total RNA was purified using Direct-zol RNA Kit (Zymo Research). Isolated total RNA was quantified and reverse transcribed using the High-Capacity cDNA Reverse Transcription Kit (Life Technologies, Cat no.4368814). The primers were arrayed in 96-well plate and were subsequently lyophilized. cDNA was prepared from 2 µg of RNA in a 20 µl reaction. For a single well, RT-qPCR was performed using 10 ng of cDNA, along with 200 nM of each primer and 7.5 µl of SYBR Select Master Mix (Life Technologies, Cat no. 4472908) in a total reaction volume of 15 µl. Cycling condition for the analysis were: 50 °C for 2 min (Hold); 95 °C for 2 min (Hold); 95 °C for 15 sec; 60 °C for 40 sec, for 40 cycles. All results were normalized against *ACTB* as the endogenous control gene. qRT-PCR was performed using QuantStudio 6 Flex Real-Time PCR System (Applied Biosystems).

For generating the heat maps, ClustVis online software was used^54^. The statistical correlation analysis function was performed using NCSS software. Pairwise matrix of correlation values was generated using heatmapper software^55^. All experiments were carried out in duplicates, and the results are presented as mean.

Sequences of the primers are proprietary. Array plate designing was done in accordance with the objectives of grant project TACR GAMA TG1010108. The primers were synthesized by Generi Biotech bearing the reference number UKLFHK-160518.

### *In vitro* differentiation (EB formation) and immunoperoxidase staining

For *in vitro* differentiation, hiPSC were cultured as floating EBs using a standard protocol^56^. These EBs were cultured for 21 days, and after this period they were fixed using 10% formalin (Lachner; Czech Republic) and embedded in paraffin. Serial sections (5–6 µm) were cut, mounted on glass slides pretreated with chrome alum-gelatin, and dried at room temperature overnight. Paraffin-embedded sections were deparaffinized by xylol (Lachner; Czech Republic) treatment, hydrated with decreasing concentrations of ethanol (Penta; Czech Republic) (96, 80 and 70%), and then rinsed twice with distilled water.

Haematoxylin and eosin staining was performed to identify various structures formed within embryoid bodies, dehydrated and mounted in DPX (Fluka).

For antigen retrieval, deparaffinized sections were exposed to microwaves (700 W) in sodium citrate solution (Lachner; Czech Republic) (pH = 6.0) twice, for 5 min each. After extensive washing with distilled water, endogenous peroxidase activity was quenched by incubating samples in 3% H_2_O_2_ (Penta; Czech Republic) (3 × 10 min). Sections were then incubated in blocking buffer (5% normal goat serum (Jackson ImmunoResearch) in PBS (Life Technologies; Cat no. 20012019)) for 20 min and exposed to primary antibodies (anti-β-III Tubulin (Exbio; Clone no: TU20), anti-MAP2 (Sigma; Clone no: HM2), anti-nestin (Life technologies; Clone no: SP103), anti-vimentin (Dako; Clone V9) and anti-pan cytokeratin (Dako; Clone AE1/AE3). Primary antibodies were diluted in Antibody Diluent with Background Reducing Components (Dako) and incubated at 4 °C overnight. After washing with PBS, sections were incubated with the following reagents: either an anti-mouse or an anti-rabbit EnVision peroxidase kit according to the manufacturer’s protocol. Colour reactions were performed using the chromogen DAB (3,3′-diaminobenzidine tetrahydrochloride, 2 µg/ml; Fluka, Darmstadt, Germany). After washing with distilled water, DAB precipitate was intensified with 3% CuSO_4_ (Penta; Czech Republic) solution for 5 min. Sections were then counterstained with Mayer’s haematoxylin (Merck) dehydrated and mounted in DPX (Fluka).

Images were captured using Olympus BX51 fluorescence microscope equipped with Olympus DP71 camera. Images were analysed and processed using Quick Photo 3.0 software.

### Directed differentiation of hiPSC to 3 lineages

#### Endodermal Lineage

hiPSC were differentiated to the endodermal lineage as described previously^57^. Briefly, the nDPSC-hiPSC, HF Eye-hiPSC and W138-hiPSC were seeded as single cells using accutase (Life Technologies) at a density of 47,500 cells/cm^2^ into Geltrex (Life Technologies) coated tissue culture plates in E8 Medium (homemade) supplemented with Rock Inhibitor Y-27632 (BOC Sciences) as described previously^58,59^. After being allowed to adhere overnight, the differentiation was initiated in order to screen for the optimal differentiation conditions for definitive endoderm (DE) formation^59,60^. Briefly, on day 0, the E8 medium was removed and the cells were washed once with DPBS without Calcium and Magnesium (DPBS^−/−^). After the wash, the cells were treated with 4 different medium combinations as follows: RPMI1640 supplemented with B27 with Insulin (Life Technologies–RB+) and additionally supplemented with 3 μM CHIR99021 (BOC Sciences); RB+ supplemented with 4 μM CHIR99021; RPMI1640 supplemented with B27 without Insulin (Life Technologies—RB−) supplemented with 3 μM CHIR99021; and RB− supplemented with 4 μM CHIR99021. On day 1, after 24 hours culture in RB with CHIR, the spent medium was changed to RB medium without any additional small molecules, and the Insulin regime was maintained the same as for day 0. On day 2, the cells were assessed morphologically for the presence of DE cells *via* microscopy analysis. The optimal conditions were then used for subsequent experiments to further differentiate the cells to hepatic endoderm (HE). For HE differentiation, the hiPSC lines were seeded as described above and the differentiation was induced using the optimal conditions determined by the first experiment (in all cases the optimal condition was RB− supplemented with 4 μM CHIR99021). After DE differentiation, the cells were treated with hepatic endoderm specification medium (SRDMSO) that was prepared and formulated as described previously^57–62^. At the HE stage, (day 7 of differentiation) the cells were collected for gene expression analysis or fixed and stained for the presence of key HE markers alpha-fetoprotein (AFP) and hepatocyte nuclear factor 4 alpha (HNF4A). Details of the primary used for immunostaining are as follows. Anti-AFP (1:500; Sigma; Cat no. A8452) and anti-HNF4A (1:100; Santa Cruz; Cat no. sc8987). Details of the secondary antibodies are as follows. Alexafluor 488 (1:400; Life Technologies; Cat no. A21206) and Alexafluor 594 (1:400; Life Technologies; Cat no. A11005).

#### Mesodermal Lineage

hiPSC were differentiated to cardiomyocytes (representing the mesodermal lineage) as described previously^63^. Briefly, cells were seeded out as single cells as described above but at different cell densities (25,000/cm^2^, 35,000/cm^2^, and 45,000/cm^2^). After the cells had adhered overnight, the cells were washed with DPBS^−/−^ and fresh E8 medium was added to the wells. The cells were cultured for an additional 2 days with medium exchanged every 24 hours. At this time point, the cells had reached between 60–90% confluence depending on initial seeding density and the differentiation commenced as described by Lian and colleagues: the concentration of CHIR99021 was tested in a range including 6, 8, 10 and 12 μM. The differentiation proceeded exactly as described by Lian and colleagues until the cells began to spontaneously contract.

#### Ectodermal Lineage

For differentiation to the ectodermal lineage, the hiPSC were differentiated to neuroepithelium using a modified version of Maroof *et al*.^64^. When hiPSC had reached 80% confluency, they were passaged at a 1:3 ratio using 0.5 mM EDTA and seeded into Geltrex coated tissue culture plates. After adhering overnight, the neural specification was commenced according to Maroof and colleagues^64^ using dual SMAD and WNT inhibition as follows. Briefly, the cells were approximately 30% confluent when starting the differentiation. The spent medium was removed and the cells were washed with DPBS^−/−^. After washing, the cells were treated with Neural Differentiation Phase I Medium consisting of Advanced DMEM/F12, 1 × Glutamax, 1 × Penicillin/Streptomycin, 1 × N2 Supplement (all from Life Technologies), and further supplemented with 10 μM SB431542 (Tocris), 100 nM LDN 193189 (Selleckchem), and 2 μM XAV-939 (Tocris). The cells were fed with this medium every 48 hours (days 0, 2, and 4). From day 5 until day 7, the medium was exchanged every 24 hours. After 7 days of differentiation, the cells were collected for gene expression analysis.

#### RT-qPCR (TaqMan)

All purchased from Life Technologies. RNA was isolated using Trizol according to the manufacturer’s instructions. cDNA synthesis was performed using the High Capacity cDNA Reverse Transcription kit (Thermo Fischer). 5ng of cDNA was used in each RT-qPCR reaction. Mastermix was the SSOAdvanced Universal Probes Supermix from Bio-rad. A 15 μl reaction volume was used. Details of Taqman probes are as follows.

*AFP -* Hs00173490_m1; *BACT* - Hs99999903_m1; *FOXG1* - Hs01850784_s1; *GATA4* - Hs00171403_m1; *HNF4A* - Hs00230853_m1; *NES -* Hs04187831_g1; *PAX6 -* Hs00240871_m1; *PROX1* - Hs0086294_m1; *SOX1 -* Hs01057642_s1

### Telomere length analysis

Genomic DNA was extracted from the cells using the DNeasy Tissue Kit (Qiagen, Germany). Telomere length measurement was performed by qPCR according to the method described by Cawthon^35^. Relative telomere length was calculated by normalizing the telomere repeat copy number to single-gene copy number and the formula used was 2^−ΔCt^ (where ΔCt = Ct_telomere_ − Ct_single-copy gene)._ 36B4, encoding ribosomal protein lateral stalk subunit P0 (RPLP0), was used as the single copy gene. Telomere and 36B4 gene PCRs were performed in separate 96-well plates with each sample run in triplicate in the same well position on an ABI 7500 HT Detection System (Applied Biosystems, USA). Each 20 μL reaction consisted of 20 ng genomic DNA, 1 × SYBR Green master mix (Applied Biosystems), and 200 nM telomere forward primer, 200 nM telomere reverse primer. Primer pair sequences used for telomere length analysis and 36B4 gene are given in Supplementary Table S2.

Cycling conditions (for both telomere and 36B4) were 10 min at 95 °C, followed by 50 cycles of 95 °C for 15 sec and 60 °C for 1 min. Following amplification, a dissociation curve was performed to confirm the specificity of the reaction. All experiments were carried out in triplicates, and the results were presented as means.

### Karyotyping

Chromosomal G-band and R-band analyses were performed at the Department of Medical Genetics, Faculty Hospital, Hradec Kralove, Czech Republic.

## Biosafety Level 2

As per biosafety guidelines, all viral transduction related experiments were performed in Biosafety Level 2 (BL-2) containment using biological safety cabinets and laminar flow hoods, and with appropriate personal safety equipment to prevent mucosal exposure/splash.

## Ethical guidelines statement

This study is based on biological material obtained for medical reasons and with the subjects’ legal (representatives) informed consent, after they have been given all the research relevant information. The extraction of the natal tooth was indicated for medical reasons. The local Ethical Committee of University Hospital Hradec Kralove ref. 200712 S01P approved the use of the dental pulp stem cells for research purposes while the usage of human platelet rich plasma was approved by Ethical Committee of the University Hospital Hradec Kralove, ref. 201011 S14P.

Human skin biopsies were obtained, with both informed donor consent and Ethical Committee approval, from donors undergoing cosmetic surgery.

Natal tooth extraction and human skin biopsies were performed in accordance with the relevant guidelines and regulations.

## Electronic supplementary material

Supplementary information

Video 1

Video 2

Video 3
