# Supplementary material for: Directed reprogramming of comprehensively characterized dental pulp stem cells extracted from natal tooth
Source: Sci Rep. 2018 Apr 18;8:6168. doi: 10.1038/s41598-018-24421-z (PMC5906561; doi:10.1038/s41598-018-24421-z)

**Directed reprogramming of comprehensively characterized dental pulp stem cells extracted from natal tooth**

**Rishikaysh. V. Pisal, Jakub Suchanek, Richard Siller,** **Tomas Soukup, Hana Hrebikova, Ales Bezrouk**, **David Kunke, Stanislav Micuda, Stanislav Filip, Gareth Sullivan and Jaroslav Mokry**

**Supplementary Table S1: Genes used for qPCR analysis.**

83 set of genes were broadly classified into three groups; pluripotency markers (52 genes), early differentiation markers (18 genes) and somatic cell markers (13 genes).

| **Pluripotency markers** | DNMT3B, PODXL, ZFP42, ACTC1, BRIX1, CD9, COL2A1, FGF4, FOXD3, GABRB3, GRB7, LEFTY1, NODAL, REST, RUNX2, TERT, ALDH1A1, ALDH2, APC, CDH2, FGFR1, HDAC2, KAT2A, KAT7, KAT8, PARD6A, COMMD3, IL6ST, LIFR, SFRP2, POU5F1, SOX2, KLF4, LIN28A, NANOG, NR5A2, UTF1, ALPL, GDF3, TDGF1, CCNA2, CDK1, CDC42, DPPA2, DPPA3, FGF2, HSPA9, MYBL2, OTX2, SOX15, TBX3, TCF3 |
| --- | --- |
| **Early differentiation markers** | PAX6, TUBB3, FGF8, BMP4, FN1, GFAP, NEUROD1, TH, GATA2, HAND1, MESP1, HBB, RUNX1, FOXA2, GATA4, HNF4A, SOX17, EOMES |
| **Somatic cell markers** | CDH1, GJB2, KRT15, NUMB, CD34, COL1A1, NCAM1, EMX2, FABP7, NES, OLG2, PECAM1, SYCP3 |

**Supplementary Table S2. Sequence of primer pairs used for determining telomere length and single copy gene 36B4.**

| **Primer name** | **Sequence of primer** |
| --- | --- |
| **Telomere forward primer** | CGG TTT GTT TGG GTT TGG GTT TGG GTT TGG GTT TGG GTT |
| **Telomere reverse primer** | GGC TTG CCT TAC CCT TAC CCT TAC CCT TAC CCT TAC CCT |
| **36B4 forward** | CAG CAA GTG GGA AGG TGT AAT CC |
| **36B4 reverse** | CCC ATT CTA TCA TCA ACG GGT ACA A |

**Supplementary Fig. S1: Immunoperoxidase staining of EB sections from nDPSC derived hiPSC for confirmation of the three germ layers.** Immunocytochemistry of (a) β-III tubulin (ectoderm marker), (b) MAP-2 (ectoderm marker), (c) nestin (ectoderm marker), (d) vimentin (mesoderm), and (e) pan-cytokeratin (endoderm). Negative control processed using the same protocol, in which primary mouse (f) or primary rabbit (g) antibody was omitted, showed no signal in cell cytoplasm; cell nuclei were counterstained with haematoxylin. Scale bar = 100 µm.


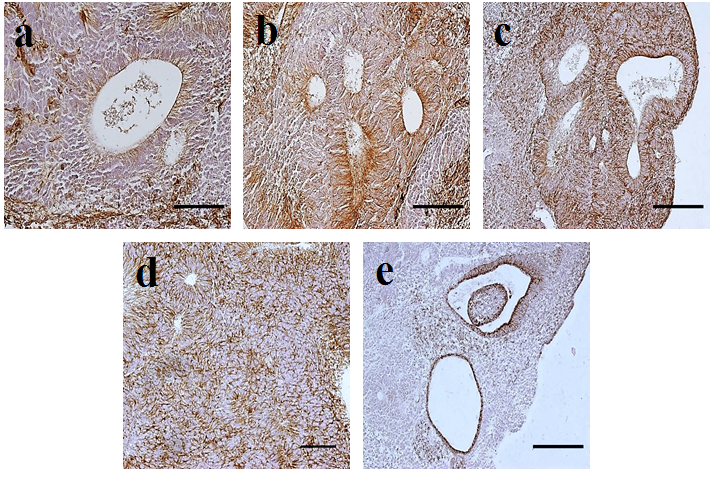


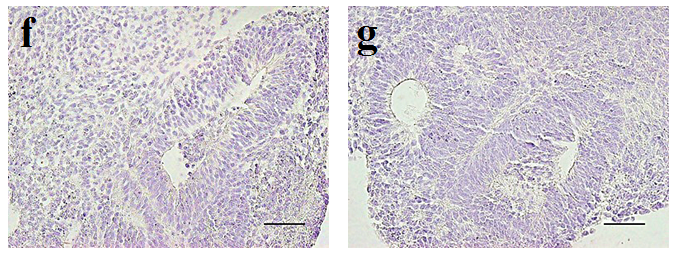


**Supplementary Figure S2. Directed differentiation of hiPSCs to the endodermal lineage - Hepatic endoderm.**

1. Directed differentiation of hiPSC to hepatic endoderm (HE). Top panel shows representative morphology of hiPSC derived definitive endoderm. Images were acquired by phase contrast microscopy. Scale bar represents 100 μm. The cell line is indicated above the column. Bottom panel shows immunostaining for the key HE markers hepatocyte nuclear factor 4 alpha (HNF4A - green) and alpha fetoprotein (AFP - red). Nuclei were counterstained with DAPI. Scale bar represents 100 μm.
2. Gene expression analysis of key hepatic endoderm (HE) markers at day 7 of directed differentiation. hiPSC derived HE was collected and analysed for expression of the key HE markers Alpha Fetoprotein (AFP), Hepatocyte Nuclear Factor 4 alpha (HNF4A), GATA4, and Prospero Homeobox 1 (PROX1). All data was normalised to each line’s respective definitive endoderm (day 2) population. Beta-Actin (BACT) was used as an endogenous control. Data is presented as the average of three biological replicates. Error bars represent the standard deviation of the three biological replicates.


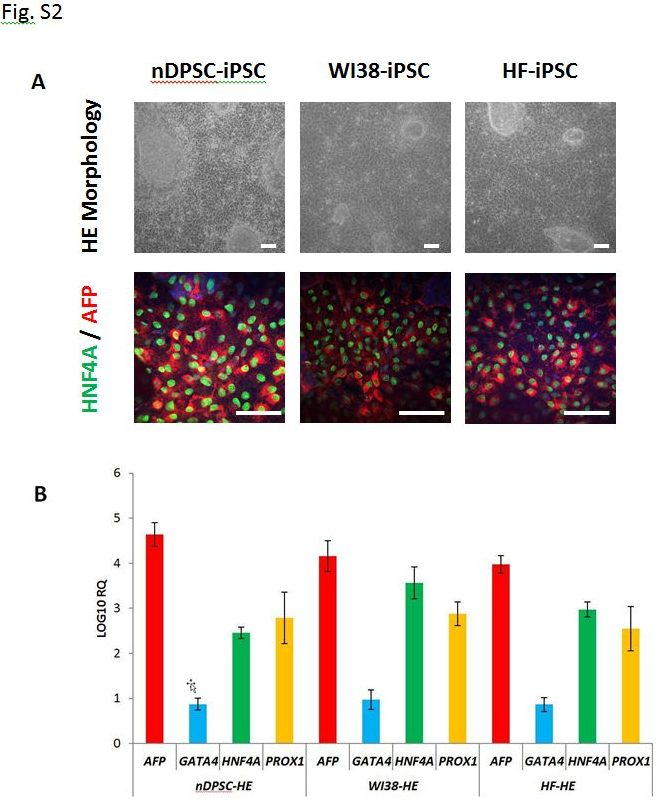


**Supplementary Figure S3. Directed differentiation of hiPSC to the ectodermal lineage -**

**Neural epithelium.**

1. Directed differentiation of hiPSC to neural epithelium (NE). Representative morphology of hiPSC derived NE after 7 days of NE differentiation. Images were acquired by phase contrast microscopy. Scale bar represents 100 μm. The cell line is indicated above the column.
2. Gene expression analysis of key NE markers after 7 days of NE differentiation. Cells were collected for gene expression analysis of the key NE markers *Forkhead Box G1 (FOXG1), Nestin (NES), Paired Box 6 (PAX6),* and *SRY (Sex Determining Region Y)-Box 1 (SOX1).* All data was normalised to each line’s respective undifferentiated hiPSC population. Beta-Actin (BACT) was used as an endogenous control. Data is presented as the average of three biological replicates. Error bars represent the standard deviation of the three biological replicates.


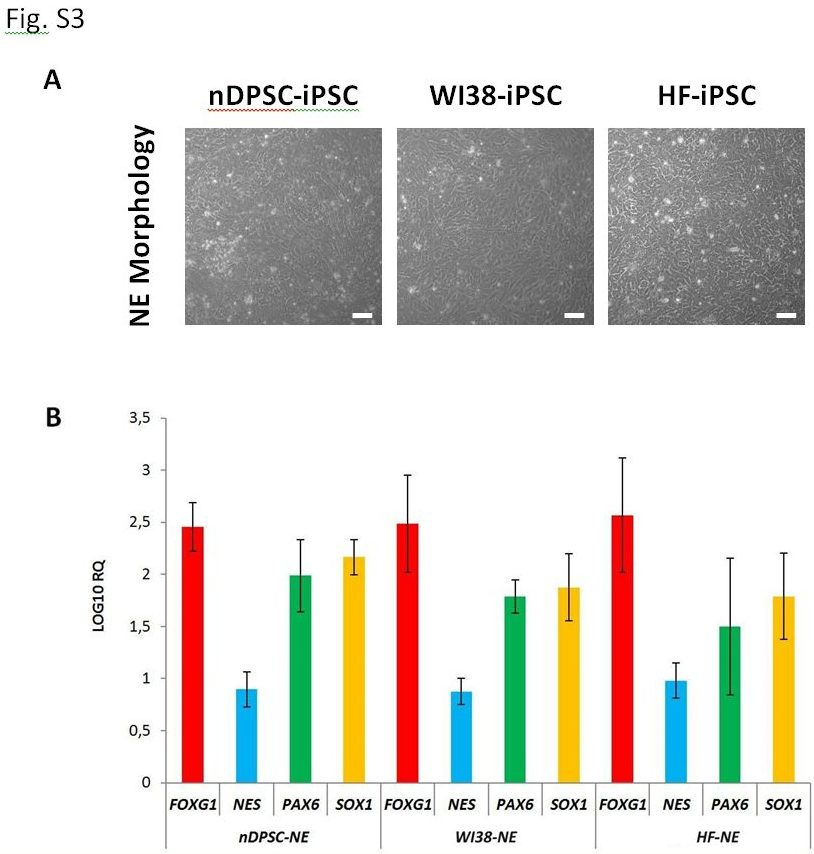


**Supplementary Video 1. Directed differentiation of nDPSC-iPSC to the mesodermal lineage - Cardiomyocytes.**

nDPSC-iPSC were differentiated to cardiomyocytes using a small molecule based approach. Beating areas were filmed using phase contrast microscopy on day 18 of differentiation.

**Supplementary Video 2. Directed differentiation of WI38-iPSC to the mesodermal lineage - Cardiomyocytes.**

WI38-iPSC were differentiated to cardiomyocytes using a small molecule based approach. Beating areas were filmed using phase contrast microscopy on day 18 of differentiation.

**Supplementary Video 3. Directed differentiation of HF-iPSC to the mesodermal lineage - Cardiomyocytes.**

HF-iPSC were differentiated to cardiomyocytes using a small molecule based approach. Beating areas were filmed using phase contrast microscopy on day 18 of differentiation.

**Supplementary Fig. S4. Karyotype analysis of reprogrammed cells.** Karyotype of nDPSC-derived hiPSC clones presented with a normal karyotype with no evidence of aneuploidy or polyploidy. The image shows clone C4.


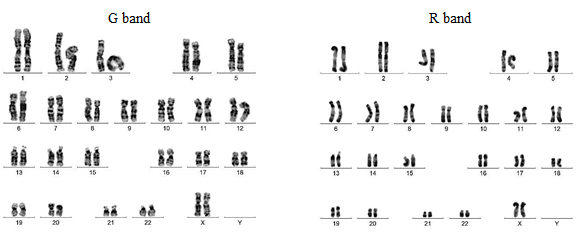

Supplement: Supplementary file 1 — Supplementary information [file 41598_2018_24421_MOESM1_ESM.doc]
